# A *Plasmodium* Promiscuous T Cell Epitope Delivered within the Ad5 Hexon Protein Enhances the Protective Efficacy of a Protein Based Malaria Vaccine

**DOI:** 10.1371/journal.pone.0154819

**Published:** 2016-04-29

**Authors:** Jairo Andres Fonseca, Monica Cabrera-Mora, Elena A. Kashentseva, John Paul Villegas, Alejandra Fernandez, Amelia Van Pelt, Igor P. Dmitriev, David T. Curiel, Alberto Moreno

**Affiliations:** 1 Emory Vaccine Center, Yerkes National Primate Research Center, Emory University, Atlanta, Georgia, United States of America; 2 Division of Infectious Diseases, Department of Medicine, Emory University School of Medicine, Atlanta, Georgia, United States of America; 3 Cancer Biology Division, Department of Radiation Oncology, Washington University School of Medicine, St. Louis, Missouri, United States of America; Swedish Neuroscience Institute, UNITED STATES

## Abstract

A malaria vaccine is a public health priority. In order to produce an effective vaccine, a multistage approach targeting both the blood and the liver stage infection is desirable. The vaccine candidates also need to induce balanced immune responses including antibodies, CD4+ and CD8+ T cells. Protein-based subunit vaccines like RTS,S are able to induce strong antibody response but poor cellular reactivity. Adenoviral vectors have been effective inducing protective CD8+ T cell responses in several models including malaria; nonetheless this vaccine platform exhibits a limited induction of humoral immune responses. Two approaches have been used to improve the humoral immunogenicity of recombinant adenovirus vectors, the use of heterologous prime-boost regimens with recombinant proteins or the genetic modification of the hypervariable regions (HVR) of the capsid protein hexon to express B cell epitopes of interest. In this study, we describe the development of capsid modified Ad5 vectors that express a promiscuous *Plasmodium yoelii* T helper epitope denominated PyT53 within the hexon HVR2 region. Several regimens were tested in mice to determine the relevance of the hexon modification in enhancing protective immune responses induced by the previously described protein-based multi-stage experimental vaccine PyCMP. A heterologous prime-boost immunization regime that combines a hexon modified vector with transgenic expression of PyCMP followed by protein immunizations resulted in the induction of robust antibody and cellular immune responses in comparison to a similar regimen that includes a vector with unmodified hexon. These differences in immunogenicity translated into a better protective efficacy against both the hepatic and red blood cell stages of *P*. *yoelii*. To our knowledge, this is the first time that a hexon modification is used to deliver a promiscuous T cell epitope. Our data support the use of such modification to enhance the immunogenicity and protective efficacy of adenoviral based malaria vaccines.

## Introduction

Malaria is the most relevant parasitic disease. Worldwide distributed, half of the human population, ~3.2 billion people, is at risk of transmission. Malaria infections account for more than 400,000 deaths every year, with 70% of the deaths occurring in children younger than five years of age [1]. The emergence, resurgence and spread of antimalarial drug resistance [2–5] along with vector resistance to insecticides [6, 7] have the potential of reducing the impact of existing malaria control measures, making a vaccine a public health priority.

Several preclinical and clinical trials have been aimed to develop an ideal malaria vaccine formulation [8]. The main challenge in designing an effective malaria vaccine is the complexity of the *Plasmodium* life cycle, as each of the different stages in the host contains a unique set of antigens that hinders the development of protective immune responses [9, 10]. Therefore, developing a multistage vaccine, able to induce strong and balanced cellular and humoral responses, is essential to obtain an effective formulation.

RTS,S/A01, the most advanced malaria vaccine candidate, is based on the *P*. *falciparum* circumsporozoite protein (CSP), a well characterized *Plasmodium* pre-erythrocytic stage antigen. In the course of phase 3 clinical trials, RTS,S/A01 showed a protective efficacy against clinical malaria of 46% in children and 27% in infants up to 18 months after vaccination [11]. The short lived efficacy could be attributed to the immunogenicity of the formulation since RTS,S/A01 induces functional antibodies but weak T cell responses [12]. Specifically, robust anti-CSP CD8+ T cells induced by immunization with RTS,S/A01 has not been reported [13], further supporting the need of balanced cellular and humoral responses.

Clinical trials with Ebola, HIV, EBV and malaria vaccines candidates have demonstrated that adenoviral (Ad) vectors are able to induce strong cellular immunity to a wide array of pathogens, while being a safe vaccine delivery system [14–19]. In the malaria model, Ad recombinant vectors have shown to induce protective cellular immune responses in heterologous prime boost regimens when boosted with a Modified Vaccinia Ankara Vector (MVA) against both hepatic [18] and blood stages [20, 21]. Nonetheless, the sterilizing protection in these studies ranged between 2 and 21%, while inducing reductions in the parasite load of the other vaccinees when compared to the control group [18, 21]. Similar results have been obtained with formulations that include a DNA prime Ad boost encoding CSP and the Apical Merozoite Antigen 1 (AMA-1) with 27% sterilizing immunity [22], a protection mainly mediated by robust CD8+ T responses [23]. Recently an immunization regimen consisting of a rare adenovirus serotype Ad35 vector, expressing the whole *P*. *falciparum* CSP without the GPI anchor, boosted with two RTS,S/AS01 immunizations showed an efficacy of 44%. Despite the high immunogenicity of this regimen, an immunization regimen consisting of three immunizations with RTS,S/AS01, had a higher efficacy of 52.4% [24]. These results, although promising, demonstrate the need for improvement in the implementation of malaria vaccine regimens incorporating novel adenoviral vectors.

A challenge that arises to implement adenoviral vectors for malaria vaccine development is the high prevalence of neutralizing antibodies against the best characterized adenoviral vector, human adenovirus serotype 5 (Ad5), especially in countries where malaria is endemic [25]. Neutralizing antibodies limit the efficacy of Ad5, even using vaccination regimens that include different Ad serotypes for boosting immunization [26]. Modifications of the adenoviral capsid, particularly the hexon protein, have been explored in malaria vaccine development to overcome pre-existing anti-vector immunity. It has been shown in the murine model that replacing the hexon hypervariable regions (HVR) of Ad5 for those of a rare adenovirus serotype can be used to circumvent pre-existing immunity against Ad5 while maintaining its immunogenicity [27]. Hexon modifications have also been used to increase the immunity against the transgene. An Ad-based malaria vaccine that expressed a B cell epitope derived from the *P*. *yoelii* CSP within the HVR 1 of Ad5 enhanced the level of protection against an experimental challenge when compared with unmodified Ad5 expressing CSP [28].

Our research group has developed a chimeric protein-based experimental vaccine derived from *P*. *yoelii* CSP and MSP-1 proteins, called *Plasmodium yoelii* chimeric multistage protein (PyCMP). PyCMP is able to induce protective CD4+ T cells and high antibody titers [29]. Our experimental vaccine includes promiscuous T cell epitopes based on orthologous sequences reported in *P*. *vivax*. These promiscuous epitopes alone have been related to protection in both the *P*. *berghei* and the *P*. *yoelii* murine malaria models [30]. Here we describe the design, production and characterization of a hexon-modified Ad5 vector that expresses the promiscuous T cell epitope PyT53 within the HVR2. This epitope is the orthologous sequence of the *P*. *vivax* promiscuous T cell epitope PvT53 identified in MSP-1 and recognized by humans with different genetic backgrounds [30, 31]. Proof-of-principle studies in mice reported here were aimed to increase the immunogenicity of our formulation and simplify the immunization regimen.

## Materials and Methods

### Design and characterization of the chimeric protein vaccine construct

The synthetic genes encoding for the *P*. *yoelii* pre-erythrocytic/erythrocytic stage antigens have been previously described [29]. A 1,242 bp synthetic Py-*cmp* gene, encoding a chimeric antigen based on the *P*. *yoelii* circumsporozoite protein genetically linked to a chimeric *P*. *yoelii* MSP-1 was transformed into *E*. *coli* BL21 (DE3) cells (Novagen, Madison, WI), and protein expression induced with 1 mM IPTG for 3 hours. The 414 amino acid hybrid protein was purified with a Ni-NTA affinity column according to the manufacturer’s instructions (Qiagen, Valencia, CA).

### Viral vectors

**Fig 1:**
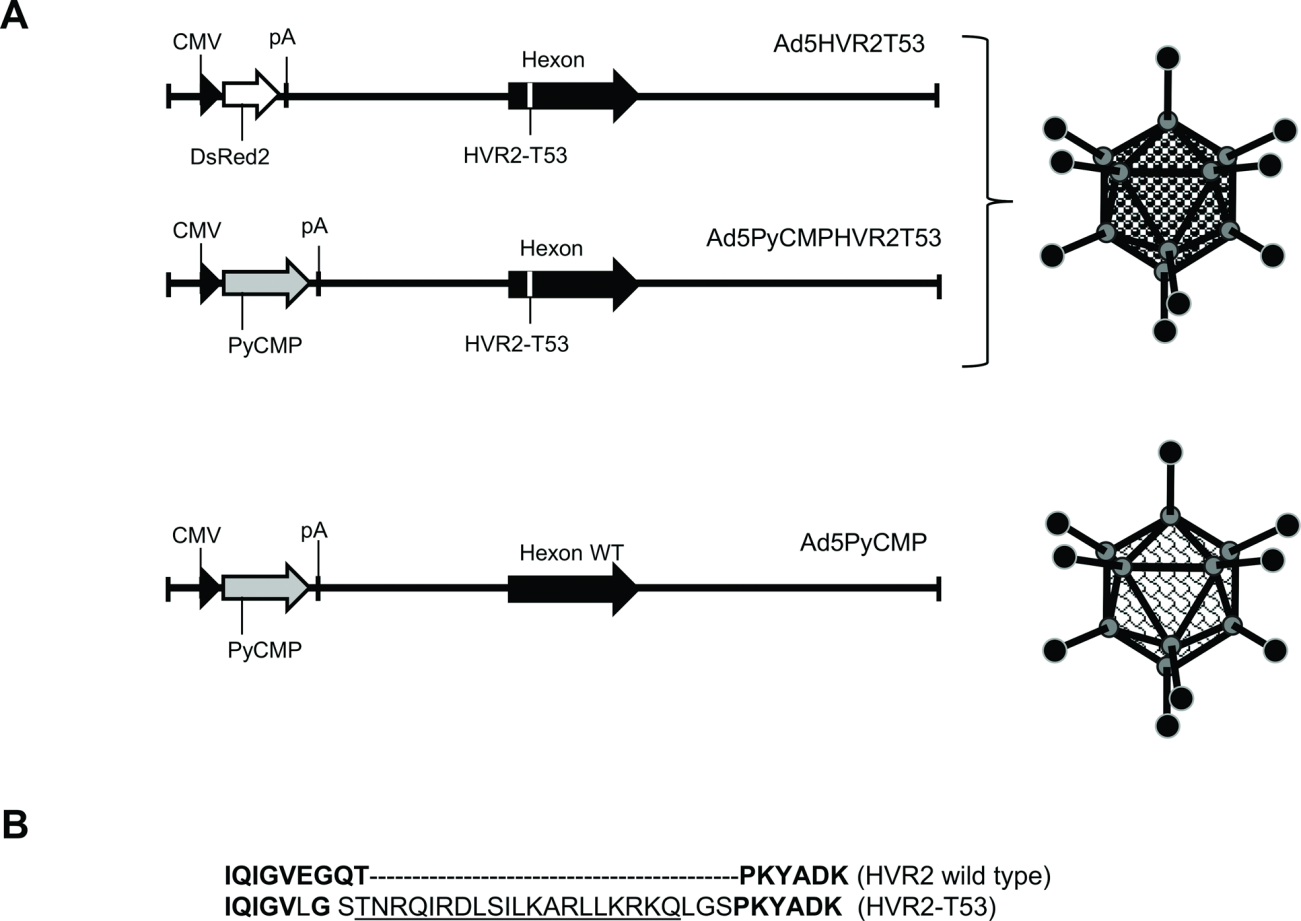
Schematic representation of the generated Ad5 vectors. **(A)** The Ad5HVR2T53 genome was constructed to incorporate the CMV promoter-driven red fluorescent *DsRed2* protein gene followed by the Simian Virus 40 (SV40) polyadenylation signal (*pA*) in place of the deleted early *E1* region. The Ad5HVR2T53PyCMP and Ad5PyCMP vectors contain the CMV promoter-driven gene encoding a *Plasmodium yoelii* chimeric multistage protein, *PyCMP*, in place of *E1*. In Ad5HVR2T53 and Ad5HVR2T53PyCMP vector, the sequence encoding hyper-variable region 2 (HVR2) of hexon gene was altered to incorporate a promiscuous *Plasmodium yoelii* T cell epitope (PyT53) **(B).** The amino acid sequence alignment showing wild type HVR2 (in bold) in the upper row aligned with HVR2 incorporating T53 epitope (underlined) in the lower row.

For adenovirus expression, the gene was codon-optimized for mammalian expression and synthesized commercially by GeneArt (Regensburg, Germany). The Py-*cmp* gene was cloned into the shuttle vector pShuttle-CMV. The resulting shuttle plasmid pCMVCMP was co-transformed with the Ad backbone E1/E3-negative plasmids, pAdEasy-1 [32], into the *E*. *coli* strain, BJ5183. The BJ5183 strain is *recA* proficient and supplies the machinery necessary for efficient homologous recombination between the shuttle plasmid and the Ad backbone plasmid. After selection on kanamycin, recombinants were screened by restriction digestion and PCR analyses for the presence of the *Py-CMP* gene insert. Positive plasmids were then transformed into a second *E*. *coli* strain, XL10-Gold (Stratagene), for amplification of the recombined Ad plasmids. The recombinant Ad plasmids were then transfected into HEK293 cells. HEK293 cells complement the E1 deletion in the Ad backbone plasmid and allow *de novo* production of Ad virions. Recombinant Ad vectors were rescued, expanded and purified by double cesium chloride sedimentation centrifugation. The physical titers, or total virus particles (VP), were determined spectrophotometrically by measuring the O.D. at 260 nm. Infectious titers were determined on HEK293 cells using a 50% end-point dilution (TCID50) assay. The expression of PyCMP was confirmed by western blot and flow cytometry analysis as described [33]. To incorporate the promiscuous CD4^+^ T cell epitope into the capsid of Ad5 we used the previously described plasmid containing Ad5 hexon DNA, modified to encode a six-histidine tag sequence (6His) flanked by BamHI and AvrII restriction sites in place of the partially deleted hyper variable region 2 (HVR2). To this end, oligonucleotide duplex encoding the *P*. *yoelii* T cell epitope TNRQIRDLSILKARLLKRKQ (PyT53) was cloned between *Bam*HI and *Avr*II sites to generate the HVR2-PyT53-containing hexon construct. This modified hexon construct was used to introduce DNA sequences encoding PyT53 within HVR2 of Ad5 genomic DNA by homologous recombination in *E*. *Coli* BJ5183 (Strategene, La Jolla, CA), as we described previously, resulting in the generation of Ad5redHVR2-T53 vectors (Fig 1). Viral genome was designed to express DsRed2 fluorescent marker protein under control of CMV promoter in place of the deleted E1 gene region. The replication incompetent Ad5redHVR2-T53 was rescued by transfecting 293 cells with the corresponding viral genomes. Modifications of the hexon gene were confirmed by PCR analysis and DNA sequencing.

The titers of physical viral particles were determined by the methods of Maizel and colleagues [34]. The titers of infectious virions were determined by plaque assay using 293 cells as described by Mittereder and colleagues [35]. The ratios of viral particles to plaque-forming units (pfu) were calculated for each recombinant Ad5 vector as follows: Ad5HVR2T53 (11.2 vp/pfu), Ad5HVR2T53PyCMP (11.7 vp/pfu), and Ad5PyCMP (10.2 vp/pfu).

### Synthetic peptides

A synthetic peptide representing the promiscuous T cell epitope PyT53 proteins was used together with a library of 58 synthetic peptides, representing the complete amino acid sequence of the hybrid protein PyCMP, for *ex vivo* stimulation. The 15-mer synthetic peptides were overlapped by 10 amino acids and commercially synthesized by Sigma-Aldrich (St Louis, MO). The peptides were dissolved in dimethyl sulfoxide and stock solutions were stored at -20°C. For *ex vivo* stimulation three peptide pools were used as described [29]: the first pool contained 14 peptides that represented the CSP sequence (Pool 1), the second pool contained 24 peptides that represented the region of the PyCMP chimeric protein that contains T cell epitopes derived from the PyMSP-1 sequence (Pool 2), and the third pool contained 20 peptides that represented the PyMSP-1_19_ sequence (Pool 3) [29]. The H-2K^d^/SYVPSAEQI/APC tetramer was synthesized at the Tetramer Core Facility (Emory University, Atlanta, GA) representing the CTL epitope of the *P*. *yoelii* CSP included in the chimeric CSP protein [30].

### Mice

Female CB6F1/J (H-2^d/b^) mice, 6 to 8 weeks of age, were purchased from Jackson Laboratory (Bar Harbor, ME). These hybrid mice were selected based on our published data concerning the response of syngeneic mice to chimeric antigens [30] and to characterize SYVPSAEQI-specific CD8+ T cells (H-2K^d^ restricted) induced by immunization. Mice were housed in micro-isolation cages and all procedures were approved by Emory University’s Institutional Animal Care and Use Committee and followed accordingly.

### Immunization regimens

Initially, 20 mice were primed intramuscularly with the Ad5HVR2T53 vector, an Ad5 vector in which its HVR2 was modified to express the *P*. *yoelii* promiscuous blood stage epitope PyT53. This vector was co-administered with 20 μg of PyCMP recombinant protein emulsified in Montanide ISA 51 (Seppic, Fairfield, NJ). Half of this group received a boosting immunization with the PyCMP recombinant protein at day 20. As controls, mice were immunized with a recombinant Ad5 expressing PyCMP as a transgene at a dose of 10^7^ vp and boosted with a PyCMP 20 days later at 20 μg or immunized with PBS in Montanide ISA 51 (Table 1). In a second experiment 20 mice received a priming immunization with 10^7^ vp of Ad5HVR2T53PyCMP or Ad5PyCMP both expressing PyCMP as a transgene followed by two boosting immunizations with 20 μg of PyCMP recombinant protein emulsified in Montanide ISA 51 at days 60 and 90 (Table 1).

**Table 1:** Immunization regimens tested with hexon modified vectors.

**Experiment 1. Co-administration regimens**					
**Priming**				**Boosting (Day 20)**	**Nomenclature**^**1**^
**Adenovirus**	**Capsid Modification**	**Transgene**	**Protein Co-administration**		
Ad5HVR2T53	HVR2^2^ –PyT53^3^	No	PyCMP^4^	PyCMP	Ad5HVR2T53+P
Ad5HVR2T53	HVR2 –PyT53	No	PyCMP	No	Ad5HVR2T53+P–P
Ad5	None	*PyCMP*	No	PyCMP	Ad5PyCMP–P
**Experiment 2. Regimens with transgene expression vectors**					
**Priming**			**Boosting (Day 60)**	**Boosting (Day 90)**	**Nomenclature**
**Adenovirus**	**Capsid Modification**	**Transgene**			
Ad5HVR2T53	HVR2 –PyT53	*PyCMP*	PyCMP	PyCMP	Ad5HVR2T53PyCMP–P–P
Ad5	None	*PyCMP*	PyCMP	PyCMP	Ad5PyCMP–P–P

1. + Indicates co-administration

- Indicates sequential Immunization.

2. Hexon Hypervariable Region 2.

3. *Plasmodium yoelii* promiscuous T cell epitope 53.

4. *Plasmodium yoelii* Chimeric Multistage Protein.

### Sporozoite challenge

*Anopheles stephensi P*. *yoelii* 17XNL infected mosquitoes were obtained from the New York University School of Medicine insectary core facility. Experimental challenges were done intravenously using 100 freshly isolated sporozoites at day 20 after the last immunization. Giemsa-stained thin smears were made daily and used to quantify parasitemia by counting the percentage of infected erythrocytes from at least 1,000 RBC per mouse using light microscopy. The procedure was performed under physical restraint equipment for the protection of both the handlers and the animals. The animals were monitored during the procedure by observing the respiratory rate and making sure that the animals were not in any distress while being in the restraint. Mice were euthanized at the end of the follow-up by carbon dioxide exposure. None of the animals died without euthanasia as a result of the experimental challenge.

### ELISA assays

The fine specificity of the antibodies elicited by immunization with the hybrid protein was determined by ELISA using Immulon 4HB plates (Thermo Labs Systems, Franklin, MA) coated with 1 μg/ml of the hybrid protein, PyCSP or PyMSP1 recombinant proteins diluted in carbonate buffer as described [36]. Optical densities were determined using a VERSAmax ELISA reader (Molecular Device Corporation, Sunnyvale, CA) with a 405 nm filter. The endpoint was measured as the highest dilution of sera that resulted in an O.D. defined by the average O.D obtained with naïve samples + 3SD. The results are presented as the reciprocal of the end-point dilution. IgG isotype profiles were also determined by ELISA as described [36]. The affinity of antibodies was assessed by a thiocyanate elution-based ELISA as described previously [31, 37].

### Flow cytometry assays

For flow cytometric analysis of the PyCMP-specific CD8^+^ and CD4^+^ T cells, peripheral blood was collected into 3.7% sodium citrate/PBS tubes and erythrocytes were lysed with ACK buffer (Life Technologies, Carlsbad, CA). After washing, the cells were incubated with α-CD3ε-PerCP (Clone: 145-2C11 Biolegend), α-CD4-Alexa Fluor 700 (Clone: RM4-5 eBioscience), α-CD11a-PerCP-Cy5.5 (Clone: M17/4 Biolegend), α-CD49d-FITC (Clone: R1-2 Biolegend), α-CD8-APC-Cy7 (Clone: 53–6.7 Biolegend), H-2K^d^/SYVPSAEQI/APC tetramer (Tetramer Core Facility, Emory University, Atlanta, GA), and α-PD1-PE Cy7 (Clone: RMP130 Biolegend) for 1 h at 4°C and then analyzed by flow cytometry. The cells were initially gated on SSC/FSC, and then the frequency of tetramer-positive cells was determined on the gated CD11a^+^CD8^+^ population (S1 Fig). The activation of CD4^+^ T cells was determined by gating the CD11a^+^ CD49d^hi^ population in CD4^+^ T cells as previously described [38].

Cellular immune responses in the spleen were measured by intracellular cytokine staining (ICS), the panel was used to simultaneously analyze IL-2, IFN-γ and TNF-α at the single-cell level in T cells derived from splenocytes obtained 10 days after the final boosting. Cells were stimulated for 6 hours with peptide pools, or with the PyCMP recombinant protein at 2 μg/ml, at 37°C in the presence of GolgiPlug (BD Biosciences). Cells were then incubated for 15 min in the presence of anti-mouse CD16/CD32 (Fc-block) before surface staining for 30 min with α-CD3ε-PerCP-Cy5.5 (145-2C11 Biolegend), α-CD8α-BV605 (Clone: 53–6.7 Biolegend), and α-CD4-Pacific Blue (Clone: GK1.5 Biolegend). Permeabilization was performed using Cytofix/Cytoperm solution (BD Biosciences) according to the manufacturer’s instructions. Cells were stained intracellularly for 30 min with α-IFN-γ-FITC (Clone: XMG1.2 Biolegend), α-IL-2-APC (JES6-5H4 Biolegend), and α-TNF-α-PE (Clone: TN3-19.12 Biolegend). All incubations were performed at 4°C. Cells were resuspended in 1% PFA solution and flow cytometry analyses were performed using an LSRII (BD Biosciences, San Jose, CA), data were analyzed using FlowJo V10 (Tree Star, Ashland, OR). The lymphocytes were initially gated on CD3^+^CD4^+^ and CD3^+^CD8^+^, then antigen-specific cytokine-secreting T cells were identified. The frequency of antigen-specific cytokine-producing cells was determined by subtracting the percentage of cytokine producing T cells after incubation with medium alone from the percentage of cytokine-producing T cells after incubation with PyCMP, or the corresponding peptide pools (S2 Fig). A threshold for a positive cytokine response was set above the background, and samples that did not meet this requirement were set to zero.

### Statistics

Statistical analysis and graphs were made using GraphPad Prism 5.0 software (GraphPad Software Inc., San Diego, CA). Antibody responses were log-transformed to achieve normality, permitting parametric testing and comparison using one-way ANOVA with Bonferoni’s post-test. For tetramer cell recognition, cellular surface markers and cytokine secreting cells, Kruskal Wallis test with Dunns post-test was used. In experimental challenges, parasitemia differences between groups were evaluated by comparing areas under the curve of parasitemia and parasitemia peak values using Kruskal Wallis test with Dunns post-test.

## Results

### 1. Capsid modified vectors, immunization regimens

We have shown that the optimal regimen for the chimeric protein PyCMP includes the use of three immunizations, as regimens containing single priming or a single boosting immunization were not protective [29]. We also have recently determined that a homologous prime-boost regimen with Ad5 expressing PyCMP as a transgene was less protective than 3 immunizations with PyCMP (manuscript submitted). These unpublished experiments also defined that protective efficacy is enhanced by using a heterologous regimen with recombinant Ad priming followed by two protein boosts immunizations.

A regimen consisting of a single or two immunizations have significant advantages for implementation in malaria endemic countries, given the limitations in primary care [39, 40]. We hypothesized that increasing the density of T cell epitopes by incorporation within the HVR2 Ad5 hexon could increase the immunogenicity of the recombinant PyCMP protein, allowing us to develop a simplified immunization regimen. We therefore initially developed an Ad5 vector that expressed a *P*. *yoelii* T cell epitope, orthologous to a well characterized CD4 promiscuous T cell epitope derived from *P*. *vivax* [30], and tested it using a co-administration with the recombinant PyCMP protein (Ad5HVR2T53+P regimen). To characterize the impact of booting immunization, an immunization regimen with a PyCMP protein boost was also tested (Ad5HVR2T53+P–P regimen). A regimen that included the recombinant Ad5 expressing PyCMP as a transgene followed by a protein boost was also tested to evaluate the need of the transgenic expression of *PyCMP* in Ad5 (Ad5PyCMP–P regimen) (Table 1).

#### 1.1. Capsid modified adenovirus antibody induction

**Fig 2:**
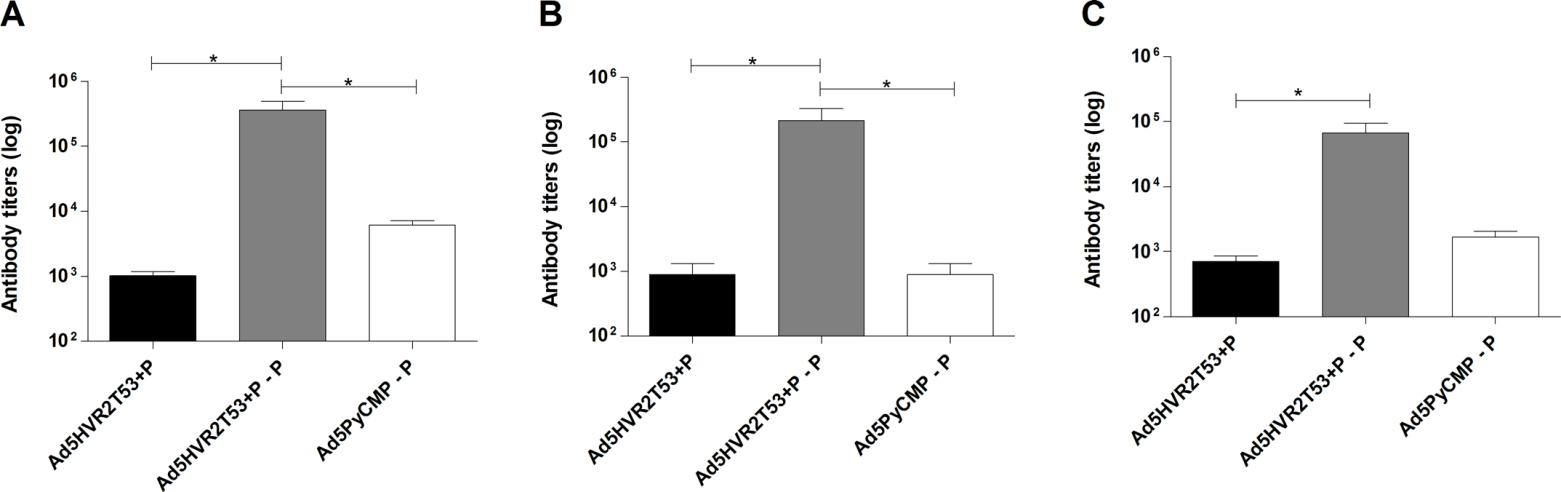
Antibody Responses induced by the vaccination regimens including Ad5HVR2 modified vectors. CB6F1/J (n = 5 per group) mice were primed with 10^7^ v.p. of Ad5HVR2T53 co-administered (+) with PyCMP a multistage *Plasmodium yoelii* protein vaccine (P) that targets a hepatic stage antigen the Circumsporozoite Protein (CSP) and a blood stage antigen the Merozoite Surface Protein 1 (MSP-1), one of the groups received a boosting immunization (-) with the protein alone. A third group received an immunization with Ad5 encoding the multistage protein as a transgene (Ad5*PyCMP*) as a prime and a boosting immunization with the multistage protein. Total IgG antibody titers 20 days after the final immunization are expressed as log against the recombinant protein **(A)**, MSP1 **(B)** and CSP **(C)**. The differences between the groups were analyzed by one-way ANOVA with a Bonferroni post-test, significant statistical differences between the groups are denoted by *(p<0.05) and ** (p<0.01).

The Ad5HVR2T53+P regimen produced an average antibody titer of 1:1,024 against PyCMP (Fig 2A), which was significantly lower when compared to the Ad5HVR2T53+P–P regimen that received a boosting immunization with PyCMP. This latter regimen induced a mean antibody titer of 1:361,472 (p<0.05). The Ad5HVR2T53+P–P regimen also produced significantly higher antibody titers against the protein when compared with the Ad5PyCMP–P regimen, which induced mean anti-PyCMP antibody titers of 1: 6,144 (p<0.05). It is important to note that when two regimens that received a protein boost were compared the antibody titers induced by the Ad5HVR2T53+P–P regimen were 59 times higher than those of the Ad5PyCMP–P group. A similar pattern of antibody distribution was observed in the different regimens when the antigens present in PyCMP (i.e. *P*. *yoelii* MSP1 or CSP) were tested in ELISA (Fig 2B and 2C). These results suggest that a protein boost is essential to induce high antibody titers and that increasing the density of T helper epitopes will have a positive effect on the B cell responses.

#### 1.2. Cellular immunogenicity induced by the capsid modified adenovirus

**Fig 3:**
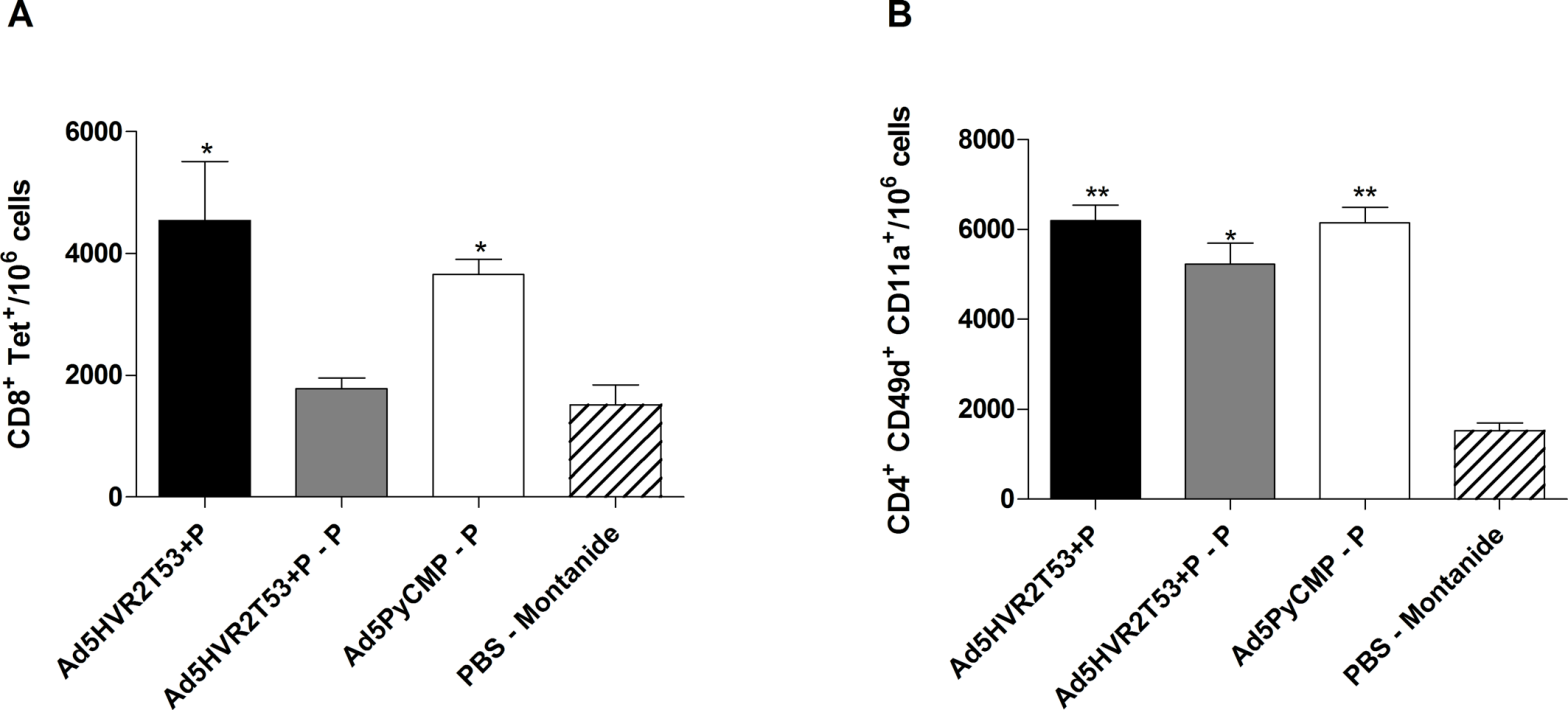
Cellular Responses induced by the vaccination regimens including Ad5HVR2 modified vectors. CB6F1/J (n = 10 per group) mice received the regimens described in Fig 2. PBMC derived from mice whole blood were obtained 5 days after the final immunization and were processed by flow cytometry, **(A)** CD8+ T cells able to recognize the *P*. *yoelii* CSP tetramer H-2K^d^/SYVPSAEQI. **(B)** CD4+ T cells expressing CD49d and CD11 as markers for the differentiation of antigen experienced cells. The results are presented as the number of cells present in 10^6^ PBMC. The differences between the groups were analyzed by Kruskal-Wallis test with Dunns post-test, significant statistical differences between the groups are denoted by *(p<0.05) **(p<0.01).

The previously reported protection induced by immunization with the chimeric protein PyCMP was associated to CD4+ T cells and antibodies [29]. To characterize the impact of immunization with the Ad vectors used, the frequency of tetramer-specific T cells was determined in PBMCs collected from immunized mice using flow cytometry 5 days after the final immunization. Both the Ad5HVR2T53+P and the Ad5PyCMP–P regimens were able to induce CD8+ T cells able to recognize the *P*. *yoelii* CSP derived H-2K^d^/SYVPSAEQI tetramer with significantly high numbers in comparison to mice that received a control immunization regimen (Fig 3A). These results indicate that the protein boost in the Ad5PyCMP–P regimen was able to maintain the levels of CD8+ T cells induced by priming with recombinant Ad5, since in the Ad5HVR2T53+P–P regimen that did not include a transgene the levels of CD8+ T cells able to recognize the tetramer after the protein boost were not significantly different than the control.

Antigen experienced CD4+ T cells are defined by their expression of CD49d and CD11a [38]. A significant expansion of CD4+CD49d+ CD11a+ T cells after the immunization with all of the regimens tested (Fig 3B) was observed. Similar to what was observed with the CD8+ T cells, the levels of antigen experienced CD4+ T cells were higher in the Ad5HVR2T53+P and the Ad5PyCMP–P regimens in comparison to the control group. However, the frequency of antigen experienced CD4+ T cells induced by the Ad5HVR2T53+P–P regimen was also significantly higher than that recorded in mice that served as controls.

#### 1.3. Cytokine production induced by the capsid modified adenovirus

**Fig 4:**
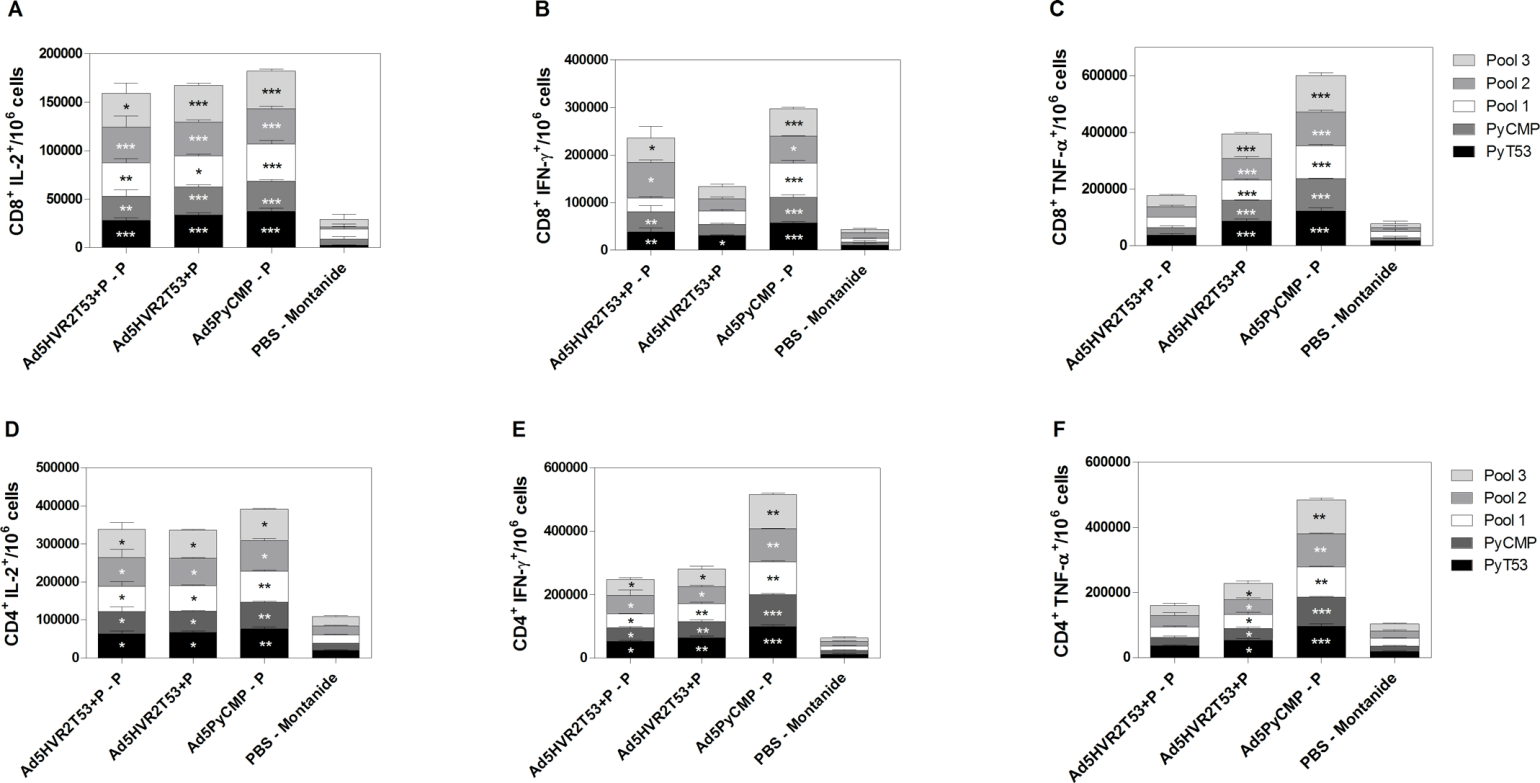
Cytokine production in splenocytes induced by the vaccination regimens including Ad5HVR2 modified vectors. CB6F1/J (n = 5 per group) mice received the regimens described in Fig 2. Splenocytes were obtained 5 days after the final immunization and were incubated with the PyT53 epitope present in the vector capsid, the PyCMP protein, or peptide pools (15 amino acids long, overlapping be 10 amino acids) representing the PyCMP sequence. After stimulation, cells were intracellularly stained and acquired by flow cytometry, **Top.** CD8+ T cells able to produce IL-2 **(A)** IFN-γ **(B)** and TNF-α **(C)** after stimulation. **Bottom.** CD4+ T cells able to produce IL-2 **(D)** IFN-γ **(E)** and TNF-α **(F)** after stimulation. Results were analyzed after background subtraction. Statistical analysis was performed using the Kruskal–Wallis test with Dunn’s post-test, differences between the vaccination groups and the control group are presented *(p<0.05) **(p<0.01) ***(p<0.001).

Splenocytes isolated five days after the final immunization were stimulated *ex-vivo* with the promiscuous T cell epitope PyT53, the PyCMP chimeric protein, a peptide pool representing PyCSP (Pool 1), a peptide pool representing the promiscuous T cell epitopes derived from the PyMSP-1 sequence (Pool 2) or a peptide pool representing the C-terminal region of the chimeric protein that comprise the PyMSP1_19_ protein fragment (Pool 3). When analyzed by flow cytometry, the CD8+ T cells produced significantly higher IL-2 with all the antigens used for *ex vivo* stimulation on every regimen when compared to control mice (Fig 4A). The Ad5PyCMP–P regimen was the best in inducing IFN-γ secreting CD8+ T cells since these cells were able to recognize all of the different antigens used for *ex vivo* stimulation. The Ad5HVR2T53+P–P regimen induced CD8+ T cells able to produce IFN-γ after *ex vivo* stimulation with all the antigens except the peptide pool representing the *P*. *yoelii* CSP. Meanwhile the Ad5HVR2T53+P regimen only induced IFN-γ secreting CD8+ T cells after *ex vivo* stimulation with the PyT53 epitope (Fig 4B). The TNF-α production by CD8+ T cells was similar in both Ad5HVR2T53+P and Ad5PyCMP–P regimens, with both being significantly higher when compared with the response from mice in the control group. However, the Ad5HVR2T53+P–P did not induce TNF-α production by CD8+ T cells (Fig 4C).

Regarding IL-2 and IFN-γ secreting CD4+ T cells were produced by all the regimens in response to all the antigens tested (Fig 4D and 4E). The frequency of TNF-α secreting CD4+ T cells showed a similar pattern to that observed for CD8+ T cells, with cells from the Ad5HVR2T53+P–P regimen unable to produce this cytokine.

#### 1.4. Protective efficacy of the capsid modified adenovirus

**Fig 5:**
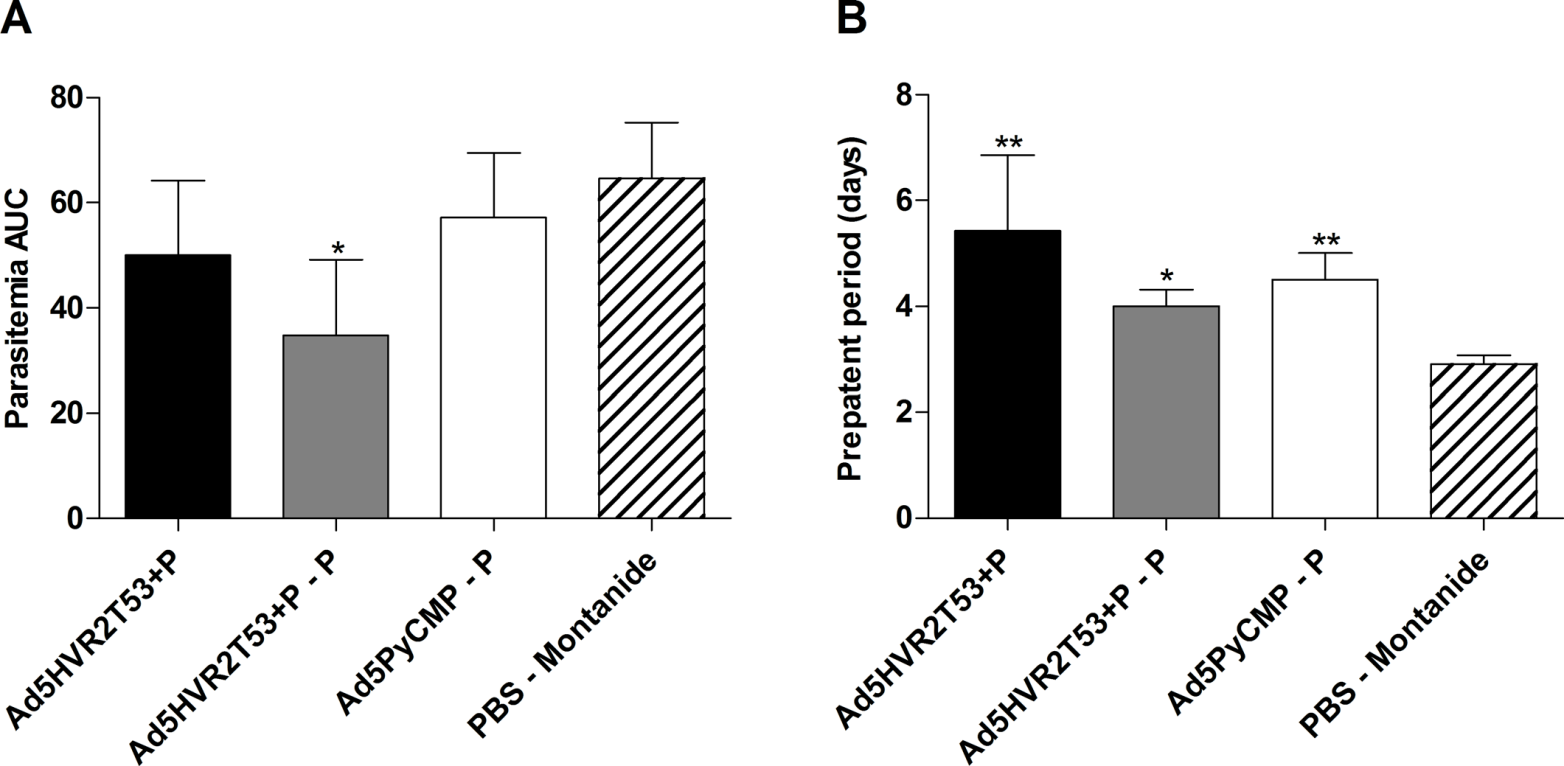
Protection induced by the vaccination regimens including Ad5HVR2 modified vectors. CB6F1/J (n = 10 per group) mice received the regimens described in Fig 2. Twenty days after the last immunizations mice were challenged with *Plasmodium yoelii* sporozoites and the kinetic of parasitemia expressed as an AUC **(A)** and the pre-patency period **(B)** were analyzed by Kruskal-Wallis test with Dunns post-test significant statistical differences between the groups are denoted by *(p<0.05) **(p<0.01).

Twenty days after the final immunization, mice were experimentally challenged with *P*. *yoelii* sporozoites. Protective efficacy was determined by comparing differences in the area under the curve (AUC) of parasitemia versus time (Fig 5A). The Ad5HVR2T53+P–P regimen was the only regimen that showed a protective efficacy with a parasitemia 1.9 times lower than the control mice (p<0.05). Although the other two immunization regimens showed no protection, the AUC analysis of parasitemia only accounts for the control of the blood stage infection. In order to define if the immunization regimens have an impact on liver stage development, the differences in the pre-patency period were analyzed. A longer pre-patency period indicates a lower parasite load in the liver, which is related to hepatic immunity [21, 41]. All of the regimens showed a significant increase in the pre-patency period when compared to control mice (Fig 5B).

### 2. Heterologous Ad-Protein immunization regimens

In the previous experiments, we demonstrated that using a modified adenovirus expressing PyT53 within the Ad5 hexon HVR2 without a transgene increases the level of antibody production when compared to the recombinant Ad5, but effective antibody titers are only obtained after protein immunization. More importantly, we learned that despite the protein boost a high and sustained IFN-γ production by both CD4+ and CD8+ T cells can only be induced if PyCMP is expressed as a transgene.

Based on this experimental evidence and our previous observations that homologous prime-boost immunization regimens with recombinant Ad vectors has lower efficacy when compared to heterologous regimens (manuscript submitted), we decided to test a capsid modified Ad5 expressing PyT53 within the hexon HVR2 and PyCMP as a transgene. The recombinant Ad5HVR2T53PyCMP vector was used for priming followed by two recombinant PyCMP boosts (Table 1). This prime-boost immunization regimen was compared to an unmodified Ad5 expressing PyCMP followed by two protein boosts (Table 1). A group of mice immunized with PBS and Montanide ISA 51 was used as a control.

#### 2.1. Antibody induction of heterologous Ad-Protein immunization regimens

**Fig 6:**
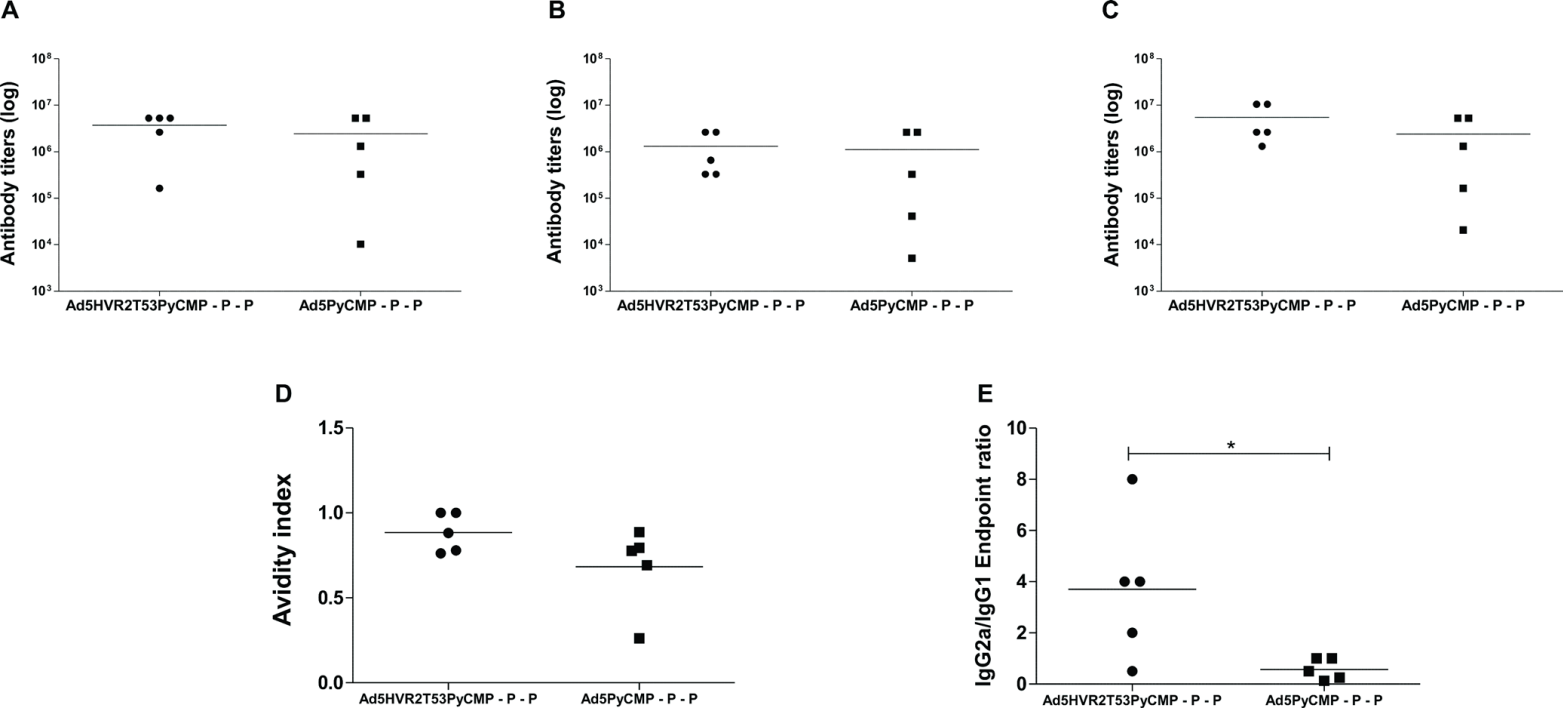
Antibody Responses induced by the vaccination regimen based on the Ad5HVRT53PyCMP vector. CB6F1/J (n = 5 per group) mice were primed with 10^7^ v.p. of Ad5 expressing the PyT53 epitope within the HVR2 hexon region and expressing PyCMP as a transgene or Ad5 expressing PyCMP. Both regimens included boosting immunizations at day 60 and at day 90 with the recombinant PyCMP protein. **Top.** Total IgG antibody titers 20 days after the final immunization expressed as log against the recombinant protein **(A)**, MSP1 **(B)** and CSP **(C)** are presented. The differences between the groups were analyzed by unpaired Student’s t test, no significant statistical differences between the groups were found. **Bottom. (D)** Affinity index of the mice sera samples after treatment with 1M NH4SCN. **(E)** IgG1 and IgG2a titers were measured separately by isotype-specific ELISA, with sera obtained 20 days after the final immunization. Data are plotted as the ratio of the endpoint log titer of IgG2a/IgG1. Differences between the groups were analyzed by Student’s t test, significant differences are denoted by *(p<0.05).

The Ad5HVR2T53PyCMP–P–P regimen produced mean anti-PyCMP titers of 1:5,505,024 while the Ad5.PyCMP–P–P regimen produced mean titers of 1: 2,396,160 (Fig 6A). Although the difference between the regimens was not significant, mice immunized with the Ad5HVR2T53PyCMP–P–P showed a lower individual variation in the response with antibody titers ranging between 1: 1,310,720 and 1: 10,485,760 while the Ad5PyCMP–P–P showed a high variability in the antibody response with antibody titers ranging between 1: 20,480 and 1: 5,242,880. As occurred with the regimens tested previously, when the anti-MSP1 and anti-CSP responses were assessed, the antibody profile was similar to the responses against PyCMP (Fig 6B and 6C).

To further characterize anti-PyCMP antibody responses, the quality of the antibodies was assessed through the antibodies avidity and evaluating the elicitation of cytophilic antibody response. The Ad5HVR2T53PyCMP–P–P regimen induced antibodies with a mean avidity index of 0.88 higher than the avidity index of 0.68 of the antibodies induced by the Ad5PyCMP–P–P regimen (Fig 6D). Interestingly, the cytophilic profile was significantly different between the regimens, with the Ad5PyCMP–P–P regimen showing an antibody production biased towards IgG1 with a mean IgG2a/IgG1 ratio of 0.6 while the Ad5HVR2T53PyCMP–P–P regimen showed a predominant IgG2a production with a mean IgG2a/IgG1 ratio of 3.7 (Fig 6E).

#### 2.2. Heterologous Ad-Protein regimens cellular immunogenicity

**Fig 7:**
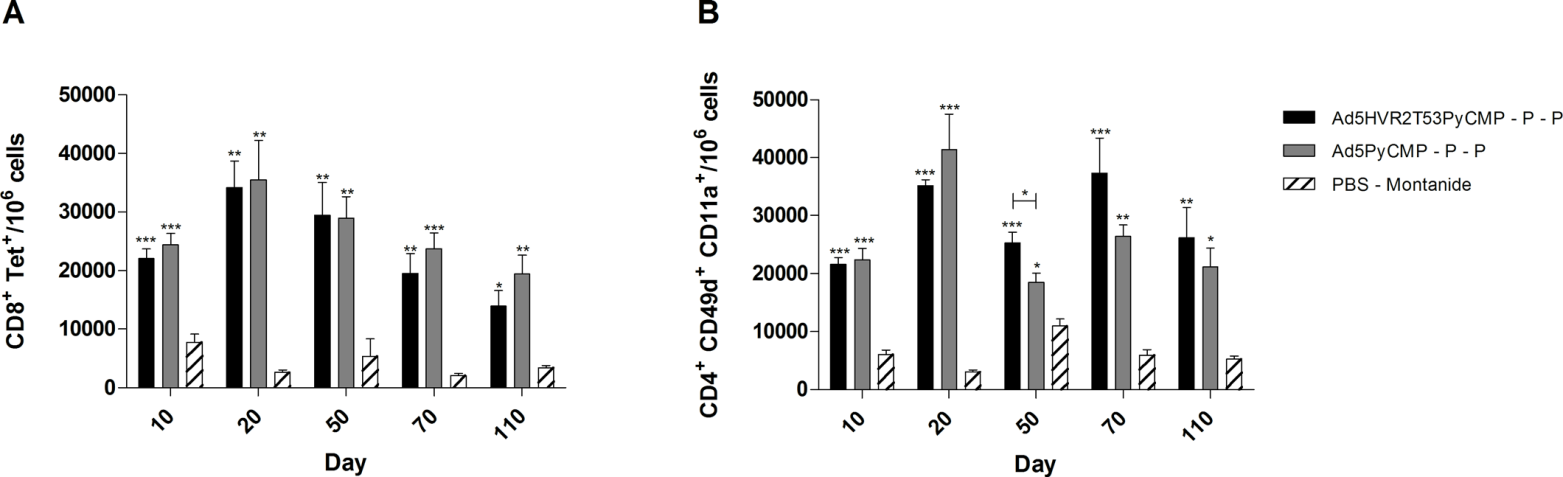
Cellular Responses induced by the vaccination regimen based on the Ad5HVRT53PyCMP vector. CB6F1/J (n = 5 per group) mice received the regimens described in Fig 6. PBMC derived from mice whole blood were obtained during the immunization schedule on the days stated in the graph and were processed by flow cytometry, **(A)** CD8+ T cells able to recognize the *P*. *yoelii* CSP tetramer H-2K^d^/SYVPSAEQI. **(B)** CD4+ T cells expressing CD49d and CD11 as markers for the differentiation of antigen experienced cells. The results are presented as the number of cells present in 10^6^ PBMC. The differences between the groups were analyzed by Kruskal-Wallis test with Dunns post-test, significant statistical differences between the groups are denoted by *(p<0.05) **(p<0.01) ***(p<0.001).

The frequency of CD8+ T cells able to recognize the *P*. *yoelii* CSP derived H-2K^d^/SYVPSAEQI tetramer induced by immunization with these regimens was analyzed at different time points in the course of immunization (Fig 7A). Both regimens were able to elicit CD8+ T cells able to recognize the tetramer with the highest numbers being 20 day after the priming. The frequency of tetramer positive T cells seems to wane overtime resulting in a frequency 36% lower in mice immunized with Ad5HVR2T53PyCMP–P–P and 21% lower in mice immunized with Ad5PyCMP–P–P at day 110 in comparison to the frequency of tetramer positive T cells obtained at day 20.

The frequency of CD4+ T cells expressing CD49d and CD11a (Fig 7B) was similar at days 10 and 20 after the priming immunization. There was a significant higher number of CD4+ CD49d+ CD11a+ T cells in the Ad5HVR2T53PyCMP–P–P regimen when compared to the Ad5PyCMP–P–P regimen at day 50 (p<0.05). An increase in the frequency of CD4+ CD49d+ CD11a+ T cells was observed 10 days after the first boosting immunization (day 70). The frequency of CD4+ CD49d+ CD11a+ T cells were 20% higher at day 110 in comparison to the numbers obtained at day 10 in the Ad5HVR2T53PyCMP–P–P regimen while in the Ad5PyCMP–P–P regimen this number was 6% lower.

#### 2.3. Heterologous Ad-Protein regimens cytokine production

**Fig 8:**
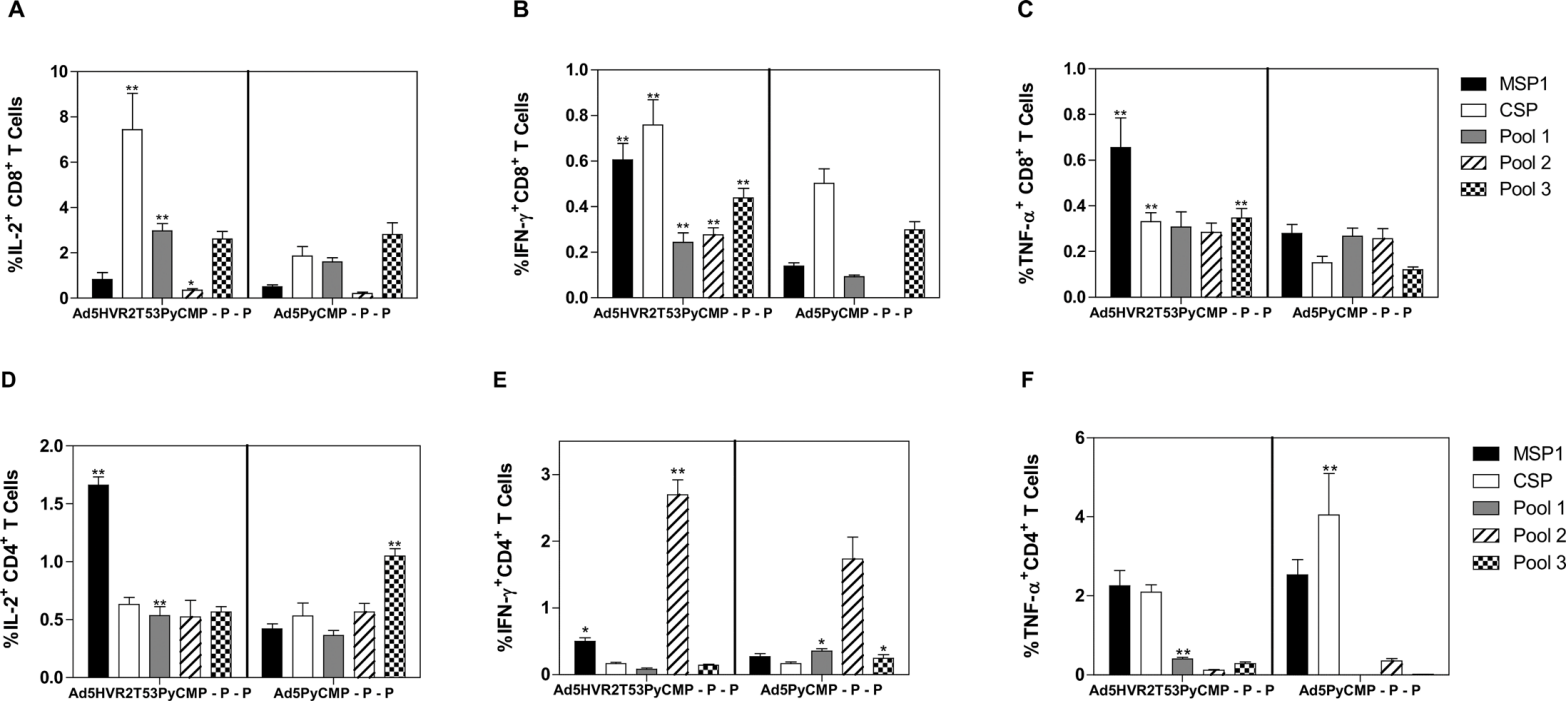
Cytokine production in splenocytes induced by the vaccination regimens based on Ad5HVR2T53PyCMP vectors. CB6F1/J (n = 5 per group) mice received the regimens described in Fig 6. Splenocytes were obtained 5 days after the final immunization and were incubated with recombinant *P yoelii* MSP1 (Black Bars) or CSP (White Bars) proteins or 15 AA overlapping peptide pools representing the PyCMP structure (Grey and Pattern Bars). After stimulation cells were intracellularly stained and acquired by flow cytometry, Results are presented after background subtraction. Top. CD8+ T cells able to produce IL-2 (A) IFN-γ (B) and TNF-α (C) after stimulation. Bottom. CD4+ T cells able to produce IL-2 (D) IFN-γ (E) and TNF-α (F) after stimulation. Differences between the immunization regimens were analyzed by the Mann-Whitney test, significant statistical differences between the groups are denoted by *(p<0.05) **(p<0.01).

The levels of cytokine producing CD8+ T cells were consistently higher in the splenocytes of mice immunized with the Ad5HVR2T53PyCMP–P–P regimen when compared to mice immunized with the Ad5PyCMP–P–P regimen. The levels of IL-2 producing CD8+ T cells were significantly higher in the Ad5HVR2T53PyCMP–P–P regimen when compared to Ad5PyCMP–P–P after the stimulation with *P*. *yoelii* CSP and the Peptide Pool encoding for this protein (p<0.01 for the protein p<0.05 for the peptide pool) The production of IL-2 by CD8+ T cells was also significantly higher when stimulated with the peptide pool containing the different promiscuous T cell epitopes present in PyCMP (p<0.01) (Fig 8A). Strikingly, the frequency of IFN-γ producing CD8+ T cells was significantly higher in the modified adenovirus regimen after *ex vivo* stimulation with all the antigens tested, suggesting a better functional T cell phenotype in mice immunized with Ad5HVR2T53 in comparison to mice immunized with the unmodified Ad5 (Fig 8B). The TNF-α producing CD8+ T cells were significantly higher in the Ad5HVR2T53PyCMP–P–P regimen when stimulated with *P*. *yoelii* CSP and MSP-1 and in the pool containing the MSP-1_19_ sequence. There were no differences in the Pool 1 representing the *P*. *yoelii* chimeric CSP and the Pool 2 representing the promiscuous T cell epitopes derived from *P*. *yoelii* MSP-1 since the recognition was high in both the Ad5PyCMP–P–P and the Ad5HVR2T53PyCMP–P–P regimens (Fig 8C). Multifunctionality of both antigen-specific CD4 and CD8 T cells was assessed using a Boolean analysis, no differences were found between the immunization regimens (data not shown).

#### 2.4. Expression of PD-1 induced by heterologous Ad-Protein regimens

**Fig 9:**
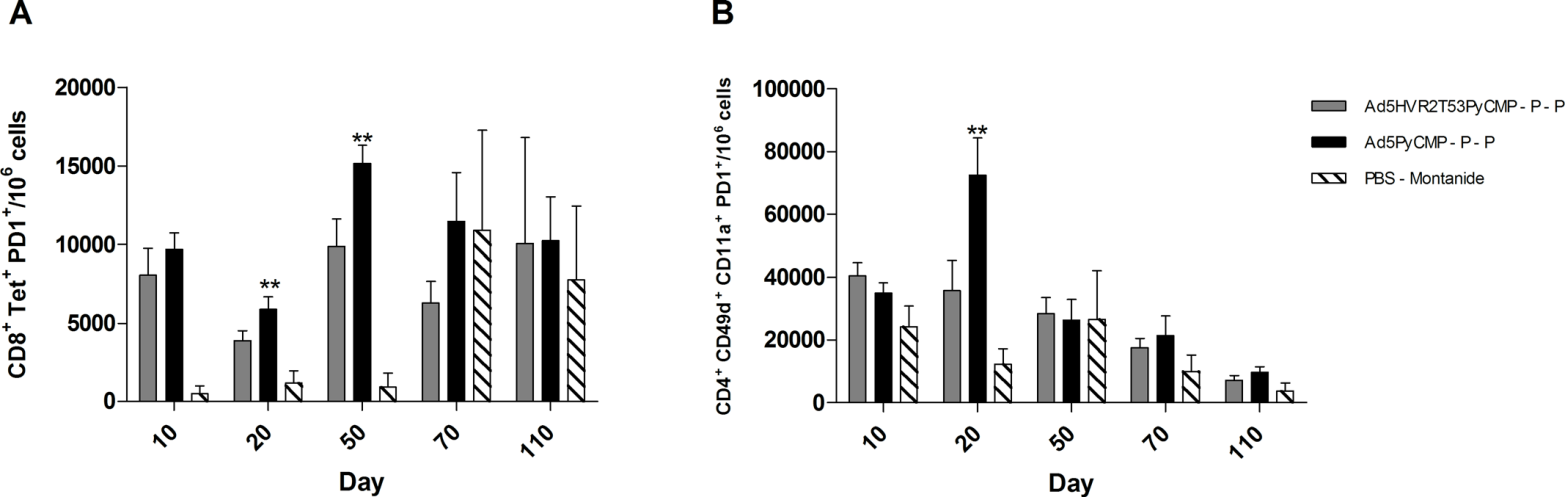
T cell PD-1 expression in PBMC induced by the vaccination regimen based on the Ad5HVRT53PyCMP vector. CB6F1/J (n = 5 per group) mice received the regimens described in Fig 6. PBMC derived from mice whole blood were obtained during the immunization schedule on the days stated in the graph and were processed by flow cytometry, **(A)** CD8+ Tetramer+ T cells PD-1 expression **(B)** CD4+ CD11+ CD49d+ T cells PD-1 expression. The results are presented as the number of PD-1 positive cells present in 10^6^ PBMC. The differences between the groups were analyzed by Kruskal-Wallis test with Dunns post-test, significant statistical differences between the groups are denoted by **(p<0.01).

To analyze whether the high density of T cell epitopes within Ad5HVR2T53PyCMP leads to T cell exhaustion, in comparison to the unmodified Ad5PyCMP, the levels of PD-1, an inhibitory receptor overexpressed in exhausted T cells was measured in antigen experienced CD4+ T cells (i.e., expressing high levels of CD11a and CD49d) and antigen specific CD8+ T cells present in PBMC cells at different time points during the immunization schedule. CD8+ T cells obtained from Ad5PyCMP immunized mice expressed significantly higher levels of PD-1 than the control at days 20 and 50 after priming (Fig 9A). In CD4+ T cells the PD-1 levels were also significantly higher after Ad5PyCMP priming at day 20 (Fig 9B). However, there were no differences between the Ad5HVR2PyCMP and the control at day 20 and 50, or between both experimental groups and the control after the protein boosts at day 60 and 90.

#### 2.5. Protective efficacy of the heterologous Ad-Protein regimens

**Fig 10:**
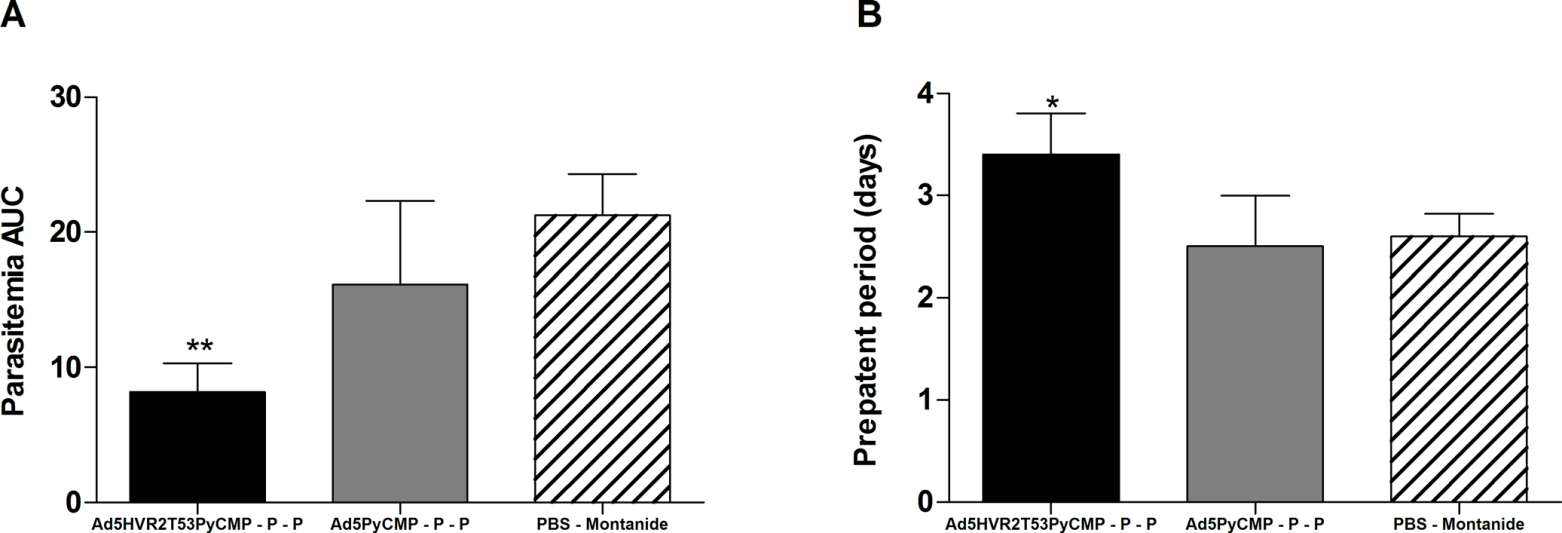
Protection induced by the vaccination regimens based on the Ad5HVRT53PyCMP vector. CB6F1/J (n = 10 per group) mice received the immunization regimens described in Fig 6. Twenty days after the last immunizations mice were challenged with *Plasmodium yoelii* sporozoites, the kinetics of parasitemia expressed as an AUC **(A)** and the pre-patency period **(B)** were analyzed by Kruskal-Wallis test with Dunns post-test, significant statistical differences between the groups are denoted by *(p<0.05), **(p<0.01).

As was done with the regimens described above, protective efficacy of the Ad5HVR2T53PyCMP–P–P and the Ad5PyCMP–P–P regimens was tested with a *P*. *yoelii* sporozoite challenge performed 20 days after the final immunization. Parasitemia was assessed as differences in the area under the curve (AUC) of parasitemia versus time (Fig 10A). The Ad5HVR2T53PyCMP–P–P regimen showed a significant reduction of the parasitemia (p<0.01) being 61.4% lower than the reported in the control group. Although the Ad5PyCMP–P–P regimen was able to reduce the parasitemia when compared to the control group in 24.0% this reduction was not significant. When the pre-patency period was analyzed, the parasitemia was significantly delayed in the AdHVR2T53PyCMP +P+P regimen, when compared to the control regimen (p<0.05). There were no differences in the pre-patency period between the Ad5PyCMP +P+P regimen and the control.

## Discussion

Novel vaccine platforms are needed to achieve the challenges imposed by the complexity of developing an effective vaccine against intracellular pathogens including malaria. Currently, RTS,S/AS01 is the only malaria vaccine candidate studied in phase III clinical trials. With boosting immunizations RTS,S/A01 achieves protection in 21% of children aged 6–12 weeks and in 30% of children aged 5–17 months [42]. The limited protection despite the immunogenicity of the formulation [13, 43] can be explained by the ability of sporozoites to evade the immune response in the liver, since the vaccine only targets the *Plasmodium falciparum* Circumsporozoite Protein (CSP) a sporozoite and early liver stage antigen, facing the possibility that a single sporozoite that evades the immune system could lead to a blood stage infection and clinical malaria [44, 45]. Therefore, a multistage malaria vaccine able to control the hepatic and the blood stage of the parasite is needed.

We developed an experimental protein-based vaccine that incorporates chimeric sequences derived from the *P*. *yoelii* CSP and the Merozoite Surface Antigen 1 (MSP1) that we called PyCMP. Proof-of-principle studies using this murine malaria parasite model showed that PyCMP is able to induce protective CD4+ T cells and high levels of functional antibodies [29]. Based on the evidence that CD8+ T cells able to recognize either the hepatic [46–48] or the erythrocytic [49] stage of *Plasmodium* are also involved in protection, we produced recombinant adenovirus vectors (Ad) that expressed PyCMP as a transgene. Ad vectors were used, as they have had been considered a suitable and safe viral vector to induce antimalarial cellular responses [18, 21, 22, 24, 50–52].

Despite being powerful inducers of cellular immune responses, the low antibody production elicited against the transgene has been considered a limitation of viral vectors. It has been recently reported that an immunization regimen that includes Ad35, a rare human adenovirus serotype, encoding the CSP C terminal region and 2 boosting immunizations with RTS,S/AS01 induced a protective efficacy of 44%, the highest protective efficacy of a malaria vaccine adenovirus regimen tested in humans. Nonetheless, the protection was still less effective than 3 immunizations with RTS,S/AS01 which induced protection in 52% of the vaccinated [24]. The lack of efficacy reported in adenoviral regimens that target blood stage antigens in malaria has been linked to the low antibody responses induce by immunization with recombinant Ads [21].

In the search of novel methods to increase the antibody production elicited by recombinant Ad vectors, capsid-modified vectors have been produced. These modifications allow the processing of the antigen by the exogenous pathway, inducing a strong humoral response [53]. Of the adenovirus capsid proteins, the most abundant is the hexon that consists of 240 trimers for a total of 720 copies per virion. Hexon represents 62.1% of the structural polypeptides by mass [54], making it an excellent target to include foreign antigens. Studies that have included antigens within the hexon, have targeted the hypervariable regions (HVR), since these sequences are not conserved and their modification do not affect the virion functionality, stability and structural integrity [55]. Of the HVR regions, HVR2 and HVR5 have shown better antigen exposure [55] and higher flexibility for incorporation of long foreign protein sequences [56].

Here we report a modification on the Ad5 hexon HVR2 by including a *P*. *yoelii* promiscuous T helper epitope denominated PyT53. This epitope is present on MSP1 and was defined based on a *P*. *vivax* orthologous sequence [30, 31]. Although most of the studies modifying the adenovirus hexon HVR have used B cell epitopes [28, 57–62], we decided to use a T helper epitope to test if the high copy number provided by the hexon modification results in the induction of a more robust and balanced cellular immune response rather than only boosting the humoral immunity. We have shown that a chimeric synthetic peptide that included the PyT53 epitope used here linked to *P*. *yoelii* B cell and cytotoxic (CTL) T cell epitopes was able to improve the antibody response and IFN-γ production when compared to immunizations with synthetic peptides representing either the B cell epitope or the CTL epitope alone [30]. PyT53 together with the B and CTL epitopes used in the previous study are also included in the PyCMP sequence.

We decided to include the PyT53 epitope within the Ad5 hexon HVR2 since our original work demonstrated the feasibility of heterologous peptide incorporation into the Ad5 HVR 2, 3, 5, 6 and 7 without significantly affecting thermostability and infectivity of hexon-modified Ad vectors [55]. We have shown that antibody binding against a hexa-histidine tag incorporated in HVR2 and HVR5 were improved as compared to HVR 3, 6, and 7 suggesting that HVR2 and 5 allow better accessibility of foreign epitopes for recognition in the context of the viral capsid. HVR2 in particular was employed to incorporate a 24 AA peptide from the HIV gp41 membrane proximal ectodomain region (MPER). Immunization with an Ad5 vector containing hexon-incorporated MPER resulted in epitope-specific humoral immune response in mice [57], suggesting that the MPER epitope is presented within the hexon in its native conformation. These findings were recently supported by cryoEM data, which revealed a variety of conformations for HVR2-incorporated MPER, including an extended form or an induced extended form amenable for recognition by a neutralizing monoclonal antibody [63]. In the aggregate, these studies provided strong rationale for incorporation of the promiscuous T cell epitope PyT53 within the HVR2.

We first tested adenoviral regimens without the transgene in order to see if the T cell epitope presentation alone was able to increase the protective efficacy of the recombinant protein PyCMP. We found that the Ad5HVR2T53+P regimen was not able to elicit antibody titers above 1x10^3^. Nonetheless, after a protein boosting the antibody titers increased to a level higher than 1x10^6^. Meanwhile, the Ad5PyCMP–P regimen induced higher antibody titers than the modified adenovirus with protein co-administration, but not as high as the co-administration with protein boost. These results indicate that the PyT53 presentation within the hexon HVR2 increases the number of CD4+ T cells able to provide help for B cells in the recognition of the chimeric PyCMP protein and the individual *P*. *yoelii* CSP and MSP1 recombinant proteins, suggesting that PyT53 can provide help to heterologous antigens as we have previously demonstrated [30, 31, 36, 64, 65]. Our results therefore confirmed that the priming of the immune system with a T helper epitope within the adenovirus capsid induces stronger antibody responses than that induced when the same epitope is presented within the transgene. It was also observed that a boost is required to obtain a high antibody response as the Ad5HVR2T53+P–P regimen had the highest antibody response. In the malaria model, our results are similar to those reported with a replicative Ad5 expressing a *P*. *falciparum* CSP B or a T/B cell epitope [66], and to those from a study using an adenovirus expressing a *P*. *yoelii* CSP B cell epitope within the HVR1 or the HVR5 and *P*. *yoelii* CSP as a transgene [28], as both regimens required between two and three boosting immunizations to induce high antibody titers [28, 66]. It is worth noting that higher Ad dosage was used in these reported studies in comparison to those tested here [28, 66]. Interestingly, Douglas et al reported a reduction in the antibody titers when a simian adenovirus AdCh63 and protein based on PfMSP1 were co-administered instead of being administered as a two-step immunization [67].

Both the Ad5HVR2T53+P and the Ad5PyCMP–P regimens, were able to induce a CD8+ T cell response able to recognize a tetramer based on a CTL epitope from the *P*. *yoelii* CSP. Importantly this CTL epitope is present in PyCMP, but it was not part of the adenovirus capsid modification. These observations suggest that the T cell epitope present in the adenovirus capsid can provide effective help for the induction of specific CD8+ T cells. An enhancement of CD8+ responses with the co-administration of Ad and protein in mice was also observed by Douglas et al [67]. In that study, the CD8+ epitope PfMSP1_42_ was expressed in the Ad vector within the transgene while the protein was PfMSP1_19_ a C-terminal fragment of PfMSP1_42_ that is poorly processed by CD8+ cells due to its structural properties [68]. The data support the idea that cognate T cell epitopes delivered within recombinant Ad vectors enhance the immune response elicited by a protein delivered in a co-administration regimen, the mechanism for this effect demands further research. Importantly, the CD8+ T cells induced by the Ad5HVR2T53+P regimen exhibit a short life, as demonstrated by a drop in the frequency of tetramer-specific CD8+ T cells in the Ad5HVR2T53+P–P regimen, a reduction not seen in the Ad5PyCMP–P regimen despite that both regimens received a protein boost. The capability of PyCMP to boost the CD8+ T cells induced by the adenovirus transgene, and not the capsid, can be explained by the long lasting expression of the transgene encoded in replication-incompetent adenoviruses as they can persist for periods longer than a year [69].

When cytokine production was evaluated only the Ad5PyCMP–P regimen was able to induce CD8+ T cells IFN-γ production in response to all of the different components of PyCMP, confirming that a transgene is necessary to induce an IFN-γ oriented CD8+ T cell response in heterologous immunization including adenoviral vectors. Our modified adenovirus was able to induce IFN-γ production by CD8+ T cells in response to PyT53. Production of IFN-γ in response to the epitopes presented in the adenovirus hexon HVR was also demonstrated by Worgall et al, using a *Pseudomonas aeruginosa* B cell epitope [62]. CD8+ IFN-γ production towards antigens present in the adenovirus HVR can be explained by the recognition of the adenovirus capsid by dendritic cells that are able to present antigens via cross-presentation, an event related to the efficacy of the responses induced by Ad-based vaccines [70, 71].

In our study, protection against a *P*. *yoelii* sporozoite challenge was analyzed using AUC of parasitemia versus time to determine the effect of immunization on the course of the blood stage infection. To analyze the immunization effect in the liver stage we used the prepatency period, as this measurement has been used as a surrogate model of parasite load and as evidence for protection against liver stages [41]. Of the immunizations tested here, the only regimen that showed an effect against both the hepatic and the blood stage was the Ad5HVR2T53+P–P regimen. This was expected, as antibodies elicited against both CSP and MSP-1have been involved in protection. In other intracellular parasites, like *T*. *cruzi*, antibodies induced by HVR modified adenovirus have also shown a protective effect [59]. Despite the low CD8+ T cell reactivity observed in the Ad5HVR2T53+P–P regimen, CD4+ T cells able to produce IFN-γ were induced by this regimen, confirming their protective role, as observed in previous PyCMP studies [29]. Protection against the liver stages was observed with the Ad5HVR2T53+P and the Ad5PyCMP–P regimens as both significantly increased the pre-patency period. The fact that these regimens did not induce protection against blood stage malaria can be explained by the poor antibody response elicited. Consistent with these results, a phase II clinical trial testing *P*. *falciparum* MSP1 and the Apical Membrane Antigen 1 (AMA1) as vaccine candidates delivered using recombinant Simian adenovirus AdCh63 and Modified Vaccinia Ankara (MVA) vectors in prime-boost immunization regimens reported an efficacy of 11% in volunteers that received vectors encoding both antigens, while none of the antigens delivered separately was able to achieve protection. This lack of efficacy was considered an effect of the low antibody response induced by the viral vectors despite their potent cellular responses [21]. Furthermore, as stated earlier, a single parasite able to escape the immune system in the liver is considered enough to produce a patent blood malaria infection.

We demonstrated that a modified adenovirus expressing PyT53 within the Ad5 hexon HVR2 without a transgene and co-administrated with PyCMP is protective since this regimen increases the level of antibody production achieving optimal titers after a protein boost. In RTS,S studies antibodies alone have been shown to be protective preventing 32% of the infections [44]. Nonetheless, clinical trials also showed that the generation of an effective cellular immune response is able to increase vaccine efficacy to 40% [44]. In this study we showed that only a transgene presentation is able to induce optimal cellular immune responses, therefore a capsid modified Ad5 expressing PyT53 within hexon HVR2 and PyCMP as a transgene seemed to be the best regimen to induce effective antibody and cellular immune responses.

To our knowledge, all of the studies involving adenovirus hexon HVR modifications have used homologous adenoviral regimens. The use of a heterologous Ad-Protein was decided, since antibodies against the foreign epitope expressed in different hexon HVR have demonstrated a neutralizing activity against the Ad infection of target cells, limiting the ability of the vector to boost the cellular responses induced by the transgene [28, 62]. Of note, a recombinant Ad5 vector with a hexon HVR2 incorporation of a 24 amino acid region of the HIV membrane proximal ectodomain region (MPER) and expressing HIV gag as a transgene was able to increase the CD8+ T cell recognition of gag after a homologous boost, but was neutralized by polyclonal Anti-Ad5 antibodies [57]. An Ad-Protein co-administration was not used given the reduction of antibody titers induced by co-administration schemes previously observed in this and other studies [67].

After the immunization with the Ad5HVR2T53PyCMP–P–P regimen the antibody titers were not significantly different that the ones obtained by the Ad5PyCMP–P–P regimen. Nonetheless, the response induced by the Ad5HVR2T53PyCMP vector was less variable than the one induced by Ad5PyCMP. It is possible that the help induced by the T helper epitope induces a better B cell reactivity, an effect seen with the PyT53 epitope and the humoral immune response against *P*. *yoelii* MSP1 [36]. This effect could be further enhanced when the same epitope is presented within the Ad capsid, since adenoviruses are able to infect Antigen Presenting Cells [72]. Moreover, Worgall et al, showed that a protein immunization with an amount equimolar to the one that would be presented by the viral particles of an adenovirus capsid presenting the same protein, induced lower humoral responses [62].

Circumventing the antigen genetic restriction could be another advantage of presenting a promiscuous T helper epitope, through an adenovirus capsid. Recently, a hexon modified adenovirus expressing a *P*. *falciparum* CSP epitope tested in monkeys reported differences in the strength of the humoral response which were attributed to the genetic diversity of the *Aotus* population [73]. We have been able to circumvent genetic restriction towards *Plasmodium* antigens both in mice [36] and humans [64, 65] using our promiscuous T cell epitopes as critical component of the vaccine construct, this could also explain the less variability in the mice receiving the Ad5PyT53+P+P regimen.

Since the antibody titers were not significantly different we decided to explore differences in the antibody profile between the Ad5HVR2T53PyCMP–P–P and the Ad5PyCMP–P–P regimens. The avidity of the antibodies induced by both regimens showed no statistical differences but was higher in the capsid modified adenovirus regimen. The development of high affinity antibodies like the one induced by the capsid modified adenovirus is encouraging as high antibody affinity against PfCSP has been related to protection using a transgenic *P*. *berghei* parasite murine model [74]. Clinical studies assessing *P*. *falciparum* blood stage antigens have also demonstrated that anti-parasite antibody dependent cellular inhibition is related to the antibody’s avidity [75]. A significantly higher IgG2a to IgG1 ratio was obtained with the hexon modified Ad regimen, although adenoviral vectors usually skew the IgG antibody responses toward cytophilic ones [26, 76, 77], the presentation of the foreign epitopes in the hexon seems to induce a better cytophilic response when compared to other adenoviral capsid regions [78]. The use of a Th1 epitope can also be responsible of this bias as Th1 cells promote the production of IgG2a [79], as seen by the high number of CD4+ T cells able to produce IFN-γ induced by our modified adenovirus. The production of cytophilic antibodies like the ones produced by the Ad5HVR2T53PyCMP–P–P regimen has been related to the inhibition of red blood cell invasion in malaria as cytophilic antibodies promote antibody mediated complement dependent immune responses [80].

Although the number of CD8+ or CD4+ T cells induced by Ad5HVR2T53PyCMP–P–P and the Ad5PyCMP–P–P regimens was not significantly different, the quality of the cellular responses was significantly better in the hexon modified adenovirus regimen inducing the production of IFN-γ and TNF-α by CD8+ T cells in response to *P*. *yoelii* CSP and MSP1 demonstrating a multistage cellular response. Importantly after the adenoviral priming the CD8+ and CD4+ T cells induced by the Ad5PyCMP priming expressed higher levels of PD-1 a marker related to T cell exhaustion [81]. The significant differences in the Th1 phenotypic response between the modified and unmodified adenovirus as observed by a lower levels cytophilic antibodies and the low IFN-γ production in the unmodified Ad5 can be explained by the PD-1 expression [82, 83]. These results are in contrast with the data reported by Teigler et al, in that study all the Ad5 HVR were replaced for those of Ad48 a rare human adenovirus serotype and both the wild type and the modified Ad5 vectors induced the expression of PD-1 and a dysfunctional phenotype when compared to Ad48 [84]. The main difference between Ad48 and Ad5, reported by the authors, is the modulation of tghe innate immune response, triggered by the different adenoviral capsid proteins [85]. The innate immune response towards the capsid and different modifications and its relation with exhausted phenotypes requires therefore further characterization.

As expected the Ad5HVR2T53PyCMP–P–P regimen induced a statistically significant protection against a *P*. *yoelii* challenge when compared to placebo, this regimen was able to reduce the parasite load both in the blood and in the liver. The Ad5PyCMP–P–P regimen was not significantly protective despite reducing the blood parasite load in 24% compared to the control group. This can be related to the higher expression of PD-1 by both CD4+ and CD8+ T cells induced by the unmodified Ad5. In the LCMV model the PD-1 expression has been related to low anamnestic potential and low protection[86]. Notably when we used the same Ad5+P+P regimen but with a shorter dose interval we obtained a high protective efficacy but when a higher adenoviral priming dose was used the protective efficacy was diminished, an effect that was linked to a lower expression of memory markers (i.e. CD62L and CD127) (Cabrera-Mora et al, submitted). Therefore, the Ad5HVR2T53PyCMP–P–P regimen has the potential to be optimized.

It can be considered that the use of Ad5 as a platform is a limitation of this study since the pre-existent immunity against this vector is between 82.2–90.5% of the population in living in malaria endemic areas [87]. Several groups have shown potential in HVR modifications to circumvent pre-existent Ad5 immunity [27, 28, 88], since the neutralizing antibodies against Ad5 are mainly directed against the hexon. The switch of Ad5 HVR for the ones of rare human serotypes has been a useful strategy, nonetheless more than one modification is usually required to achieve this effect [27, 89], since the modification of a HVR that induces high humoral responses is not necessarily related to the evasion of pre-existent anti-adenoviral antibodies [90]. We therefore consider that the promiscuous T cell epitope HVR incorporation should be performed on rare human serotypes or simian adenovirus that are not affected by the pre-existent Ad5 immunity [25, 91, 92], a strategy currently under development by our group.

In summary, in this study we generated an Ad5-based adenovirus vector that expresses a *Plasmodium* promiscuous T cell epitope within the HVR2 of the hexon capsid protein and a multistage protein vaccine as a transgene. When this vector was used in a heterologous Ad-protein immunization regimen it was able to induce a better humoral and cellular immune response in comparison to an Ad5 vector expressing the same transgene. The better immunological profile of the capsid modified vector regimen translated into a higher protective efficacy against a murine malaria challenge. The optimization of this antigen capsid incorporation will include the use of both simian and rare human serotypes adenoviral vectors to develop more attractive vectors for clinical development.

## Supporting Information

S1 Fig Gating strategy for flow cytometry analysis, tetramer. In this sample gating, cells were first gated for lymphocytes (SSC-A vs FSC-A) and then for singlets (FSC-H vs FSC-A). The singlets gate was further analyzed for CD3 expression taking only the T cell population (CD3^+^). CD4 and CD8 surface expression was then determined and the CD8^+^ T cells were further analyzed to measure tetramer recognition.
(PDF)

S2 Fig Gating strategy for flow cytometry analysis, ICS. In this sample gating, cells were first gated for lymphocytes (SSC-A vs FSC-A) and then for singlets (FSC-H vs FSC-A). The singlets gate was further analyzed for their uptake of the Alexa 430 Live/Dead Stain. The samples were then analyzed by gating on the live population and CD3^+^ T cells selected for further characterization of CD4^+^ and CD8^+^ T cell subsets. IFN-γ, TNF-α and IL-2 producing CD4^+^ or CD8^+^ T cells were then quantified.
(PDF)
