# Supplementary material for: A Plasmodium Promiscuous T Cell Epitope Delivered within the Ad5 Hexon Protein Enhances the Protective Efficacy of a Protein Based Malaria Vaccine
Source: PLoS One. 2016 Apr 29;11(4):e0154819. doi: 10.1371/journal.pone.0154819 (PMC4851317; doi:10.1371/journal.pone.0154819)
Supplement: S1 Fig — In this sample gating, cells were first gated for lymphocytes (SSC-A vs FSC-A) and then for singlets (FSC-H vs FSC-A). The singlets gate was further analyzed for CD3 expression taking only the T cell population (CD3+). CD4 and CD8 surface expression was then determined and the CD8+ T cells were further analyzed to measure tetramer recognition. (PDF) [file pone.0154819.s001.pdf]

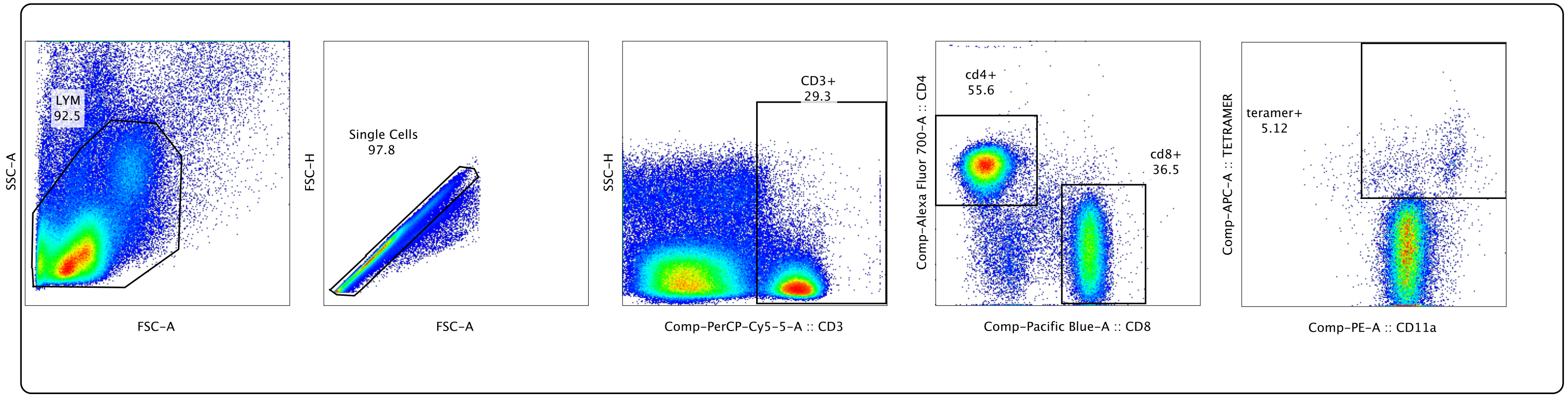

S1Fig. Gating strategy for flow cytometry analysis, tetramer. In this sample gating, cells were first gated for lymphocytes (SSC-A vs FSC-A) and then for singlets (FSC-H vs FSC-A). The singlets gate was further analyzed for CD3 expression taking only the T cell population (CD3+). CD4 and CD8 surface expression was then determined and the CD8+ T cells were further analyzed to measure tetramer recognition.
