# Supplementary material for: A Plasmodium Promiscuous T Cell Epitope Delivered within the Ad5 Hexon Protein Enhances the Protective Efficacy of a Protein Based Malaria Vaccine
Source: PLoS One. 2016 Apr 29;11(4):e0154819. doi: 10.1371/journal.pone.0154819 (PMC4851317; doi:10.1371/journal.pone.0154819)
Supplement: S2 Fig — In this sample gating, cells were first gated for lymphocytes (SSC-A vs FSC-A) and then for singlets (FSC-H vs FSC-A). The singlets gate was further analyzed for their uptake of the Alexa 430 Live/Dead Stain. The samples were then analyzed by gating on the live population and CD3+ T cells selected for further characterization of CD4+ and CD8+ T cell subsets. IFN-γ, TNF-α and IL-2 producing CD4+ or CD8+ T cells were then quantified. (PDF) [file pone.0154819.s002.pdf]

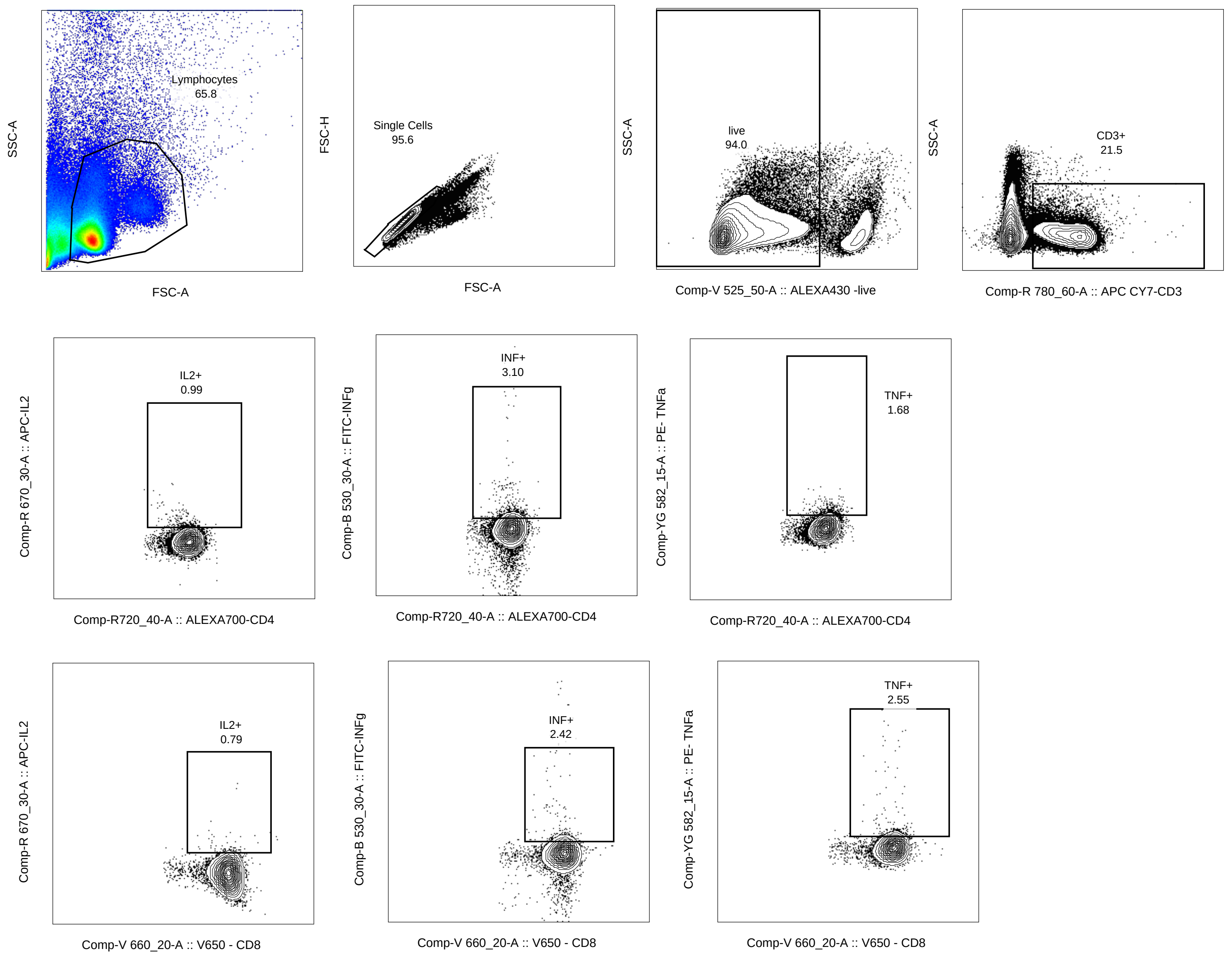


**S2 Fig. Gating strategy for flow cytometry analysis, ICS.** In this sample gating, cells were first gated for lymphocytes (SSC-A vs FSC-A) and then for singlets (FSC-H vs FSC-A). The singlets gate was further analyzed for their uptake of the Alexa 430 Live/Dead Stain. The samples were then analyzed by gating on the live population and CD3+ T cells selected for further characterization of CD4+ and CD8+ T cell subsets. IFN-γ, TNF-α and IL-2 producing CD4+ or CD8+ T cells were then quantified.
